# Publisher Correction: Tailoring of an unusual oxidation state in a lanthanum tantalum(IV) oxynitride via precursor microstructure design

**DOI:** 10.1038/s42004-020-0280-7

**Published:** 2020-03-06

**Authors:** Cora Bubeck, Marc Widenmeyer, Gunther Richter, Mauro Coduri, Eberhard Goering, Songhak Yoon, Anke Weidenkaff

**Affiliations:** 1 Institute for Materials Science, University of Stuttgart, Heisenbergstraße 3, 70569 Stuttgart, Germany; 2 Materials and Resources, TU Darmstadt, Alarich-Weiss-Straße 2, 64287 Darmstadt, Germany; 3 Central Scientific Facility Materials, Max Planck Institute for Intelligent Systems, Heisenbergstraße 3, 70569 Stuttgart, Germany; 4 European Synchrotron Radiation Facility (ESRF), 71 Avenue des Martyrs, 38000 Grenoble, France; 5 Modern Magnetic Systems, Max-Planck-Institute for Intelligent Systems, Heisenbergstraße 3, 70569 Stuttgart, Germany; 6 Fraunhofer IWKS, Rodenbacher Chaussee 4, 63457 Hanau, Germany

**Keywords:** Solid-state chemistry, Synthetic chemistry methodology, Design, synthesis and processing

Correction to: *Communications Chemistry* 10.1038/s42004-019-0237-x, published online 26 November 2019.

The original version of this Article incorrectly omitted the received/accepted dates of Received: 28 March 2019; Accepted: 24 October 2019. This has now been corrected in the PDF and HTML versions of the Article.

